# COVID-19 vaccination carries no association with childbirth rates in Sweden

**DOI:** 10.1038/s43856-026-01396-x

**Published:** 2026-01-21

**Authors:** Dennis Nordvall, Thomas Schön, Jorma Hinkula, Olle Eriksson, Armin Spreco, Örjan Dahlström, Johan Lyth, Daniel Axelsson, Elin Gursky, Marie Blomberg, Toomas Timpka

**Affiliations:** 1 Region Jönköping County, Jönköping, Sweden; 2 Linköping University, Linköping, Sweden; 3 William Carey University, Hattiesburg, MS USA

**Keywords:** Epidemiology, Epidemiology, Preventive medicine

## Abstract

**Background:**

Speculative claims about COVID-19 vaccines affecting fertility and childbirth have circulated widely. We aimed to examine whether COVID-19 vaccination is causally associated with childbirth in Swedish women.

**Methods:**

We conducted a cohort study in a Swedish total population of 369,000 to emulate an experiment comparing childbirth rates between 59,773 vaccinated and unvaccinated women aged 18–45 years. Cox proportional hazards models were applied, treating vaccination as a time-varying covariate. Causal modeling was used to adjust for potential bias. To capture vaccine effects on both conception and established pregnancies, the index event was set at an estimated conception date, 280 days prior to childbirth.

**Results:**

We show that with an assumed average pregnancy length of 280 days, there are no statistically significant associations between COVID-19 vaccination and childbirth (unadjusted HR = 0.94 (95% CI 0.89-1.00); adjusted HR = 1.03 (95% CI 0.97-1.09)). Assuming a shorter pregnancy length (266 days), the associations between vaccination and childbirth remain insignificant (unadjusted HR = 0.96 (95% CI 0.90-1.02); adjusted HR = 1.04 (95% CI 0.98-1.11)). Neither are there statistically significant associations between COVID-19 vaccination and recorded miscarriages (unadjusted HR = 0.84 (95% CI 0.69-1.03); adjusted HR = 0.86 (95% CI 0.70-1.05)).

**Conclusions:**

COVID-19 vaccination is not associated with a decrease in childbirth after adjusting for common confounding factors. These findings provide evidence to support vaccination policies for women of childbearing age.

## Abstract (web summary)

Nordvall et al. use a cohort study design to study causal effects of the COVID-19 vaccine on childbirth rates in a representative Swedish population. They show that COVID-19 vaccination has no association with either childbirth rates or miscarriages.

## Plain Language Summary

Early, unfounded rumors spread during the COVID-19 pandemic claimed that mRNA vaccination could cause infertility. Later, suspicions were raised regarding whether reductions in childbirth observed during the pandemic were associated with the novel COVID-19 vaccines. We therefore study effects of COVID-19 vaccination in a representative population of Swedish women, and adjust for any confounding effects. No association is observed between COVID-19 vaccination and childbirth, or between vaccination and recorded miscarriages. We thus find no evidence for connections between COVID-19 vaccination and the observed decrease in childbirth. Our results are relevant for consideration when vaccination policies involving women of childbearing age are determined.

## Introduction

Speculation regarding the side effects of COVID-19 vaccines on childbirth rates is widely disseminated on social media^1^. An early rumor spread during the pandemic claimed that mRNA vaccine could cause infertility by inducing antibodies that bind to a placental protein. However, further investigations showed that there were no data to support this mechanism^2^. Later, suspicions were raised regarding whether reductions in childbirth observed during the pandemic were associated with the novel COVID-19 vaccines^3^. Epidemiological studies have not identified negative associations between fetal development or preterm birth and receipt of COVID-19 vaccination administered during any trimester^4–6^. Also population-level associations between COVID-19 vaccine roll-out and total childbirth rates have been investigated, mainly by examining of correlations between time series of aggregated data from national public registers. In the Czech Republic, successful conceptions, that is, conceptions leading to live births 9 months later, have been compared for women who were either vaccinated or unvaccinated against COVID-19^7^. The rates of successful conception were found to be lower among vaccinated women than in those who were not vaccinated. Another study covering 26 high-income countries observed a negative association between having received the first dose of the COVID-19 vaccination and total childbirth rates, whereas having completed the two-dose vaccination course was linked to a recovery of birth rates^8^. In a similar study, a negative association between COVID-19 vaccination and birth rates was reported from 10 of 22 high-income countries, but only in a few nations did the childbirth rates decline to a level below the predicted long-term trajectory^9^.

Although there is no research evidence that COVID-19 vaccines affects fertility or childbirth rates, misleading information on social media is requiring systematic debunking^10^. The evidence regarding COVID-19 vaccines has also not been sufficiently persuasive with policymakers^11^.

We aimed to investigate associations between COVID-19 vaccination and childbirth decrease during the pandemic using health registry data from a representative Swedish population (*N* = 369,000) employing causal modeling for corrections to bias. Based on assumptions about causal associations and confounding bias, we emulated an experiment where COVID-19 vaccine was randomly distributed to women of childbearing age on 1 January 2021 for determining differences in childbirth propensity in individuals receiving and not receiving the vaccine.

The results show that COVID-19 vaccination is not associated with childbirth decrease after correcting for common sources of bias. This finding should be considered when deciding vaccination implementation policies for women in childbearing age.

## Methods

A cohort study design was used including data on all women 18-45 years of age on 1 January 2021 in Jönköping County, Sweden, (*N* = 59,773). Data on childbirths (ICD-10 O80-O84), miscarriages (ICD-10 O03), COVID-19 vaccinations, and deaths were collected from the healthcare provider Region Jönköping County and Statistics Sweden from 1 January 2016 to 31 December 2024. Data on induced abortions were not available for ethical reasons. Basic COVID-19 vaccination was made available to county residents aged ≥18 years from January 2021 by provision of 2 doses Comirnaty (Pfizer-BioNTech), Spikevax (Moderna) or the Vaxzevria (AstraZeneca) vaccine. Booster doses were administered beginning September 2021. An informed consent waiver was provided by the Swedish Ethical Review Authority (EPM 2023-05203-02) upon approval of the study design and ethical vetting consistent with Swedish legislation permitting the use of healthcare databases for research.

### Analysis model

**Fig. 1:**
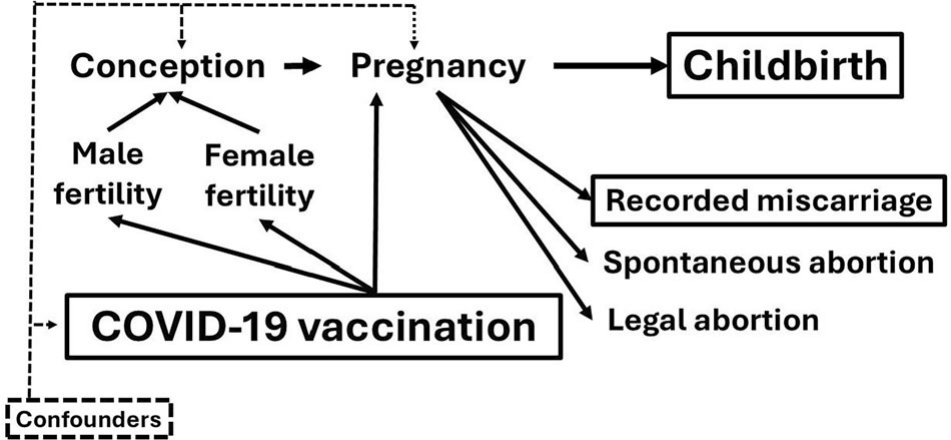
Conceptual model for analysis of causal associations between COVID-19 vaccination and childbirth showing pathways for confounding bias. Data on boxed factors is contained in standard health registries while information on the unboxed factors is not routinely covered.

Directed acyclic graphs were used to develop a conceptual model outlining assumptions about causal relationships and confounding structures between the exposure, COVID-19 vaccination, and the outcome, childbirth (Fig. 1). Based on this framework, age and comorbidity were identified as potential confounders. Ongoing comorbidity was assumed to be associated with (a) a higher propensity to accept vaccination and (b) a greater likelihood of voluntarily abstaining from pregnancy. Adjustment for comorbidity was therefore expected to attenuate any negative association between vaccination and childbirth, and the analyses consequently adjusted for age only.

### Statistical analyses

Cox proportional hazards models were applied using the second dose of COVID-19 vaccine as exposure and childbirth as outcome. The outcome (index) event was selected to ensure that vaccination could influence both conceptions and established pregnancies; the date for the index event was therefore moved from the date of childbirth to an estimated conception date 280 days earlier. The index event was accordingly defined as the estimated conception for a recorded childbirth 280 days later. Data from childbirths (ICD-10 O80-O84) recorded between 8 October 2021 and 6 July 2024 were accordingly used for the analyses. Censoring events were third dose (booster) vaccination, death, and end of follow-up. The propensity of childbirth relative to vaccination adjusted for age was estimated using Hazard Ratio (HR). Violation of the proportional hazard assumption was assessed by inspection of survival curves and the Schoenfeld test.

Restricting pregnancy identification to childbirths precludes accounting for pregnancies ending in spontaneous abortion. An estimated 12-15% of recognized conceptions result in miscarriage^12^. We therefore conducted a separate analysis to examine the association between COVID-19 vaccination and recorded miscarriages (ICD-10 O03). Pregnant women were followed in the health information system from the clinical visit at which pregnancy was confirmed by a healthcare practitioner. Early spontaneous abortions were not captured unless women sought healthcare, resulting in incomplete ascertainment of pregnancy losses. Consequently, complete data on both recognized and unrecognized conceptions were not available in standard health registries. From gestational week ≥22 + 0, pregnancy losses were recorded as stillbirths. To avoid misclassification, women recorded as being ≥22 + 0 weeks pregnant on 1 January 2021 were excluded from this analysis.

To establish whether the estimate of pregnancy length and thereby assumed conception date influenced the study conclusion, a sensitivity analysis was performed by changing the average length of pregnancy to 266 days. An additional sensitivity analysis of a potential healthy vaccinee effect was conducted of incomplete vaccinations (<2 doses) using one dose of the COVID-19 vaccine as basic exposure to assess alternative exposure definitions.

All statistical analyses were performed using the R software package version 4.3.0.

## Results

**Fig. 2:**
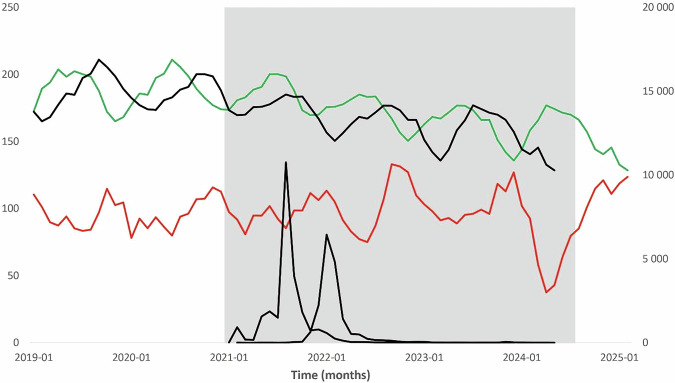
Conceptions leading to childbirth (left y-axis), recorded miscarriages (left y-axis), and COVID-19 vaccinations (right y-axis) in Jönköping County, Sweden, January 2019 to January 2025. Upper black curve - estimated conceptions leading to childbirth per 100,000 women (moving average); Green curve - childbirth per 100,000 women (moving average); Red curve - recorded miscarriages per 1000 childbirths (moving average); Lower black curves - dose 2 and 3 vaccinations per 100,000 women; Grey shadowed area – study period.

The basic two-dose COVID-19 vaccination was received by 45,165 (75.5%) of the 59,773 women aged 18-45 years included in the study (Table S1); 791 women (1.3%) received only one vaccine dose. Ninety-seven percent of the vaccine doses were of the mRNA type (Comirnaty, Spikevax). In the vaccination period, childbirths in Jönköping County decreased by 8% from 2021 to 2022, 4% from 2022 to 2023, and 3% from 2023 to 2024 (Fig. 2, Table S2). Ten percent (*n* = 5955) of the women gave birth before receiving a booster vaccination dose. One percent (*n* = 542) of the women recorded as not ≥20 weeks pregnant on 1 January 2021 had a recorded miscarriage during the vaccination period, corresponding to 91 miscarriages per 1000 childbirths.

### Associations between COVID-19 vaccination and childbirth

With an assumed average pregnancy length of 280 days, we found no statistically significant association between COVID-19 vaccination and childbirth (unadjusted HR = 0.94 (95% CI 0.89–1.00); adjusted HR = 1.03 (95% CI 0.97–1.09). No violation of the proportional hazard assumption was found by inspection of survival curves (Figure S2.1) or application of the Schoenfeld test (Figure S3.1).

### Robustness of findings

Assuming a shorter pregnancy length (266 days), the association between vaccination and childbirth remained insignificant (unadjusted HR = 0.96 (95% CI 0.90–1.02); adjusted HR = 1.04 (95% CI 0.98–1.11)). No statistically significant association was observed between vaccination and recorded miscarriages (unadjusted HR = 0.84 (95% CI 0.69–1.03); adjusted HR = 0.86 (95% CI 0.70–1.05)) (Table S4). Correspondingly, when defining vaccine exposure as receipt of at least one vaccine dose (assuming a 280-day pregnancy duration), the association between vaccination and childbirth remained statistically insignificant (unadjusted HR = 0.93 (95% CI 0.88–0.99; adjusted HR = 1.02 (95% CI 0.96–1.09)). For recorded miscarriages, the corresponding hazard ratios also remained insignificant (unadjusted HR = 0.84 (95% CI 0.70–1.00); adjusted HR = 0.90 (95% CI 0.74–1.09)) (Table S5).

## Discussion

We adjusted our analyses for confounding by accounting for age-related variation in childbirth and vaccination propensities. Vaccination acceptance is known to be lower among women who are knowingly pregnant than among their non-pregnant peers; that is, pregnancy itself may lead women to abstain from vaccination^13^. To address this potential reverse causality, we additionally shifted the index event from childbirth to the estimated date of conception. However, these adjustments did not account for conceptions ending in spontaneous abortion, leaving the analysis susceptible to selection bias. Although such bias could in principle be addressed by weighing individuals by the inverse of their probability of selection^14,15^, this approach was not feasible due to the lack of reliable data on the probabilities of recognized and unrecognized conceptions across different levels of exposure and outcome. While selection bias associated with early spontaneous abortions is not a concern in studies restricted to confirmed pregnancies^16^, estimating the total effect of vaccination on childbirth in the general population requires explicit consideration of this issue. We therefore conducted a separate analysis examining associations between vaccination and recorded miscarriages. This analysis showed a non-significant trend toward a reduced likelihood of spontaneous abortion among vaccinated women, consistent with evidence identifying COVID-19 infection as a risk factor for pregnancy loss^17^. Taken together, our findings indicate that COVID-19 vaccination was not associated with a decrease in childbirth during the pandemic, under the assumption that vaccine effects on documented and undocumented pregnancy losses are similar. To reduce selection bias in future obstetric vaccine safety studies, health registries could be enhanced by incorporating menstrual health data collected outside clinical settings^18^.

There are alternative and more plausible explanations for the decline in childbirths observed in the study population than COVID-19 vaccination, including pandemic-related socio-economic conditions and behavioral changes associated with lockdowns^9^. Moreover, official demographic statistics show an increase in annual childbirths from 1983 to around 1990 in Sweden, followed by a decline between 1992 and 1998^19^. During the mid- to late 1990s, the country experienced strained public finances and reductions in social support for families with children^20^. Given that the median age of parents of children born between 2021 and 2024 was approximately 31 years (Table S1), the pool of potential parents was already shrinking because of lower birth rates three decades earlier. Socio-economic conditions and sociodemographic determinants therefore represent the most plausible explanations for the observed decrease in childbirth rates during the pandemic.

## Supplementary information

Transparent Peer Review file

Supplementary materials

Description of Additional Supplementary files

Supplementary Data

## Data Availability

The dataset of this study is the property of the health service providers involved and was provided to the researchers through a restricted-access agreement that prohibits sharing the dataset with third parties or making it publicly available. Access to the data is restricted to preserve the confidentiality of patient information and was granted to researchers for research purposes only. Individuals or entities interested in accessing the data may contact professor Toomas Timpka via email at toomas.timpka@liu.se. All proposed research must obtain the necessary ethical approvals. No datasets, whether raw or de-identified, can be publicly released by the researchers. However, aggregate data that do not compromise individual privacy are included within the manuscript and supplementary materials. This ensures transparency of the research findings and supports the reproducibility of results while maintaining compliance with legal requirements. Source Data for Fig. 2 is provided as Supplementary Data.

## Code availability

The R code required to replicate our results is available from GitHub at 10.5281/zenodo.18138478.
