# Supplementary material for: COVID-19 vaccination carries no association with childbirth rates in Sweden
Source: Commun Med (Lond). 2026 Jan 21;6:360. doi: 10.1038/s43856-026-01396-x (PMC13310814; doi:10.1038/s43856-026-01396-x)
Supplement: Supplementary file 2 — Supplementary materials [file 43856_2026_1396_MOESM2_ESM.pdf]

Supplementary Materials

Contents

Page 2 Table S1 Basic COVID-19 vaccinations (2 doses) 2021-2024 in women residing in Jönköping County, Sweden

Page 3 Table S2 Annual births per 1000 women and year 2016 to 2024 in women residing in Jönköping County, Sweden

Page 4 Table S3 Annual miscarriages per 1000 women and year 2016 to 2024.

Page 5 Table S4 Summary of main results.

Page 6 Figure S1 Extended display of childbirth trend 2016-2025 in Jönköping County, Sweden.

Page 7 Figure S2.1 and S2.2 Survival curves comparing vaccinated and unvaccinated women aged 18-45 years in Jönköping County, Sweden with respect to childbirth and miscarriage 2021-2023.

Page 8-9 Figure S3.1 and S3.2 Schoenfeld tests of proportional hazards assumption.

Page 10 Figure S4 HR(t) for childbirth (280 days pregnancy) based on natural cubic splines.

Page 11 Table S5 Additional sensitivity analyses associated with vaccine dose 1.

Table S1 Basic COVID-19 vaccinations (2 doses) 2021-2024 in women residing in Jönköping County, Sweden, displayed by age group month. N=59,773.

Age<br>Month	18-25 years	26-30 years	31-35 years	36-40 years	41-45 years	All
2021-01	2	6	5	8	12	33
2021-02	294	270	296	372	427	1659
2021-03	64	57	65	71	78	335
2021-04	51	54	76	57	57	295
2021-05	579	563	531	540	592	2805
2021-06	566	590	652	629	880	3317
2021-07	408	347	386	479	956	2576
2021-08	2705	2868	4443	4356	4599	18971
2021-09	2790	2001	1076	802	501	7170
2021-10	811	740	754	564	383	3252
2021-11	406	394	226	171	120	1317
2021-12	421	352	291	194	148	1406
2022-01	322	247	209	170	96	1044
2022-02	124	120	81	70	38	433
2022-03	61	59	46	32	18	216
2022-04	16	21	14	15	5	71
2022-05	21	18	9	8	10	66
2022-06	15	13	16	13	4	61
2022-07	6	6	9	6	2	29
2022-08	4	4	3	2	0	13
2022-09	7	2	8	3	2	22
2022-10	3	2	4	2	1	12
2022-11	1	2	2	0	1	6
2022-12	2	2	0	1	1	6
2023-01	1	1	5	2	1	10
2023-02	0	0	1	2	0	3
2023-03	3	1	2	0	1	7
2023-04	3	2	0	3	0	8
2023-05	1	1	1	0	0	3
2023-06	1	1	0	0	1	3
2023-10	0	1	1	0	0	2
2023-11	0	2	2	0	1	5
2023-12	0	1	1	0	1	3
2024-02	0	1	1	0	0	2
2024-11	0	0	2	1	0	3
2024-12	0	0	0	1	0	1
Total	9688	8749	9218	8574	8936	45165

Table S2 Annual births per 1000 women and year 2016 to 2024 in women aged 18-45 years residing in Jönköping County, Sweden, displayed by age group and year. The median age for childbirth in 2021 was 30.5 years (mean age 30.7 years).

Age<br>Year	18-25 years	26-30 years	31-35 years	36-40 years	41-45 years	All
2016	865	1681	1087	459	88	4180
2017	842	1623	1103	462	76	4106
2018	811	1719	1181	448	47	4206
2019	695	1718	1195	427	62	4097
2020	682	1698	1263	460	57	4160
2021	598	1625	1315	425	75	4038
2022	522	1417	1277	454	80	3750
2023	514	1386	1182	411	69	3562
2024	473	1271	1246	447	55	3492
Total	6002	14138	10849	3993	609	35591

Table S3 Annual miscarriages per 1000 women and year 2016 to 2024 by age category.

**Age**<br>**Year**	**18-25 years**	**26-30 years**	**31-35 years**	**36-40 years**	**41-45 years**	**All**
2016	94	157	127	73	26	477
2017	87	133	127	65	29	441
2018	95	129	141	77	23	465
2019	57	136	117	54	23	387
2020	56	146	110	64	27	403
2021	46	121	118	74	27	386
2022	50	115	137	67	16	385
2023	51	107	99	60	33	350
2024	44	91	73	58	20	286
Total	580	1135	1049	592	224	3580

Table S4 Summary of main results.

	**Model 1. (N=56,787)**	**Sensitivity Model 1. (N=56,935)**	**Model 2. (N=59,661)**
	Childbirth (280 days pregnancy)	Childbirth (266 days pregnancy)	Miscarriage (within 22 weeks)
unadjusted	0.94 (0.89-1.00)	0.96 (0.90-1.02)	0.84 (0.69-1.03)
adjusted for age	**1.03 (0.97–1.09)**	**1.04 (0.98-1.11)**	**0.86 (0.70-1.05)**
0-212 days	0.99 (0.71-1.40)	0.91 (0.75-1.10)	1.23 (0.69-2.22)
213-365 days	0.95 (0.86-1.05)	0.98 (0.88-1.09)	0.82 (0.54-1.27)
366-1074 days	1.11 (0.97-1.28)	1.11 (1.02-1.20)	0.82 (0.65-1.05)

Figure S1 Extended display of childbirth trend 2016-2025 in Jönköping County, Sweden. Lower dashed graphs show receipt of second (black) and third (red) doses of COVID-19 vaccine.

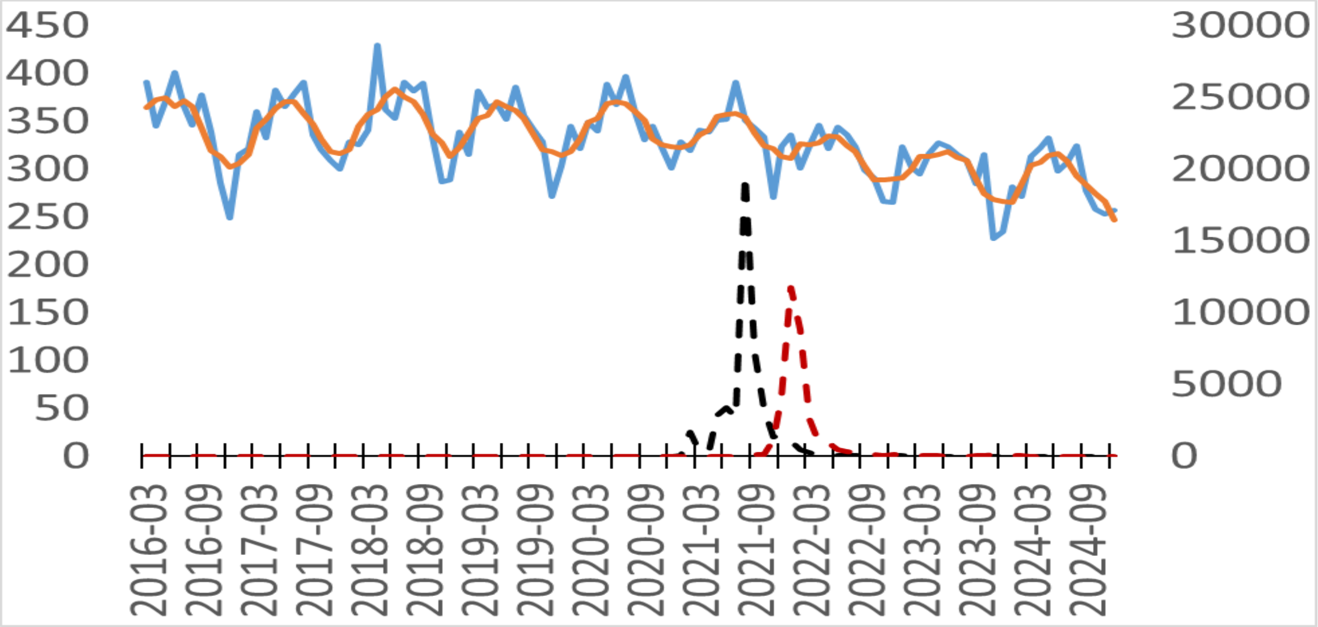

Figure S2.1 Survival curves adjusted by age vaccinated (exposure=1) and unvaccinated (exposure=0) women aged 18-45 years in Jönköping County, Sweden with respect to the outcome childbirth [time scale in days].

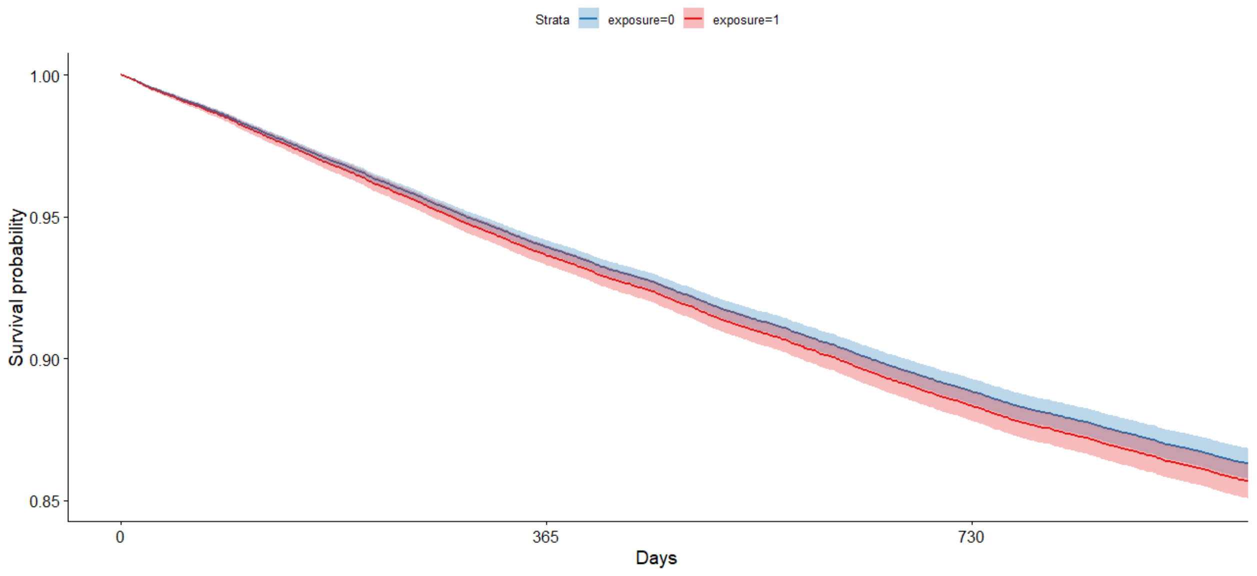

Figure S2.2 Survival curves adjusted by age and comparing vaccinated (exposure=1) and unvaccinated (exposure=0) women aged 18-45 years in Jönköping County, Sweden with respect to the outcome miscarriage [time scale in days].

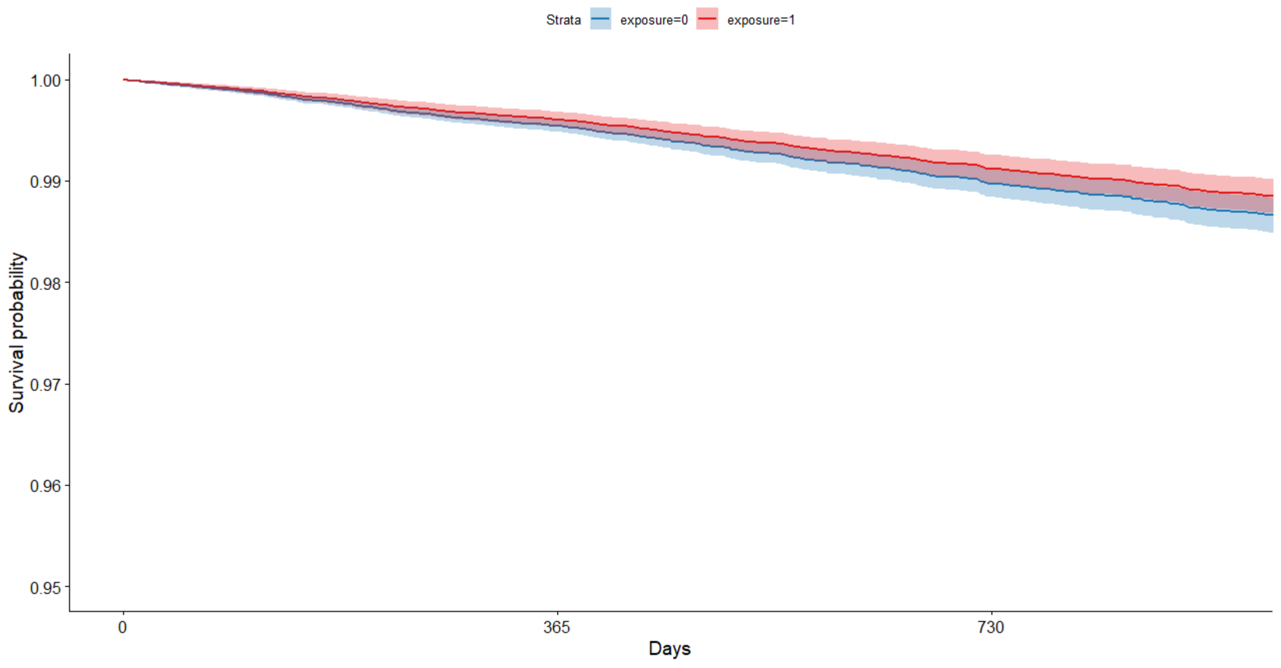

Figure S3.1 Schoenfeld tests of proportional hazards assumption for Childbirth. Residuals are scaled and plotted against time. Separate tests are performed for the three periods (*Period 1* Vaccination of risk groups and healthcare workers, *Period 2* Mass vaccination, and *Period 3* Follow-up vaccination of unvaccinated individuals) defined by the two knots in the Cox proportional hazard model.

Period 1 Vaccination of risk groups and healthcare workers 2021-01-01 to 2021-07-31

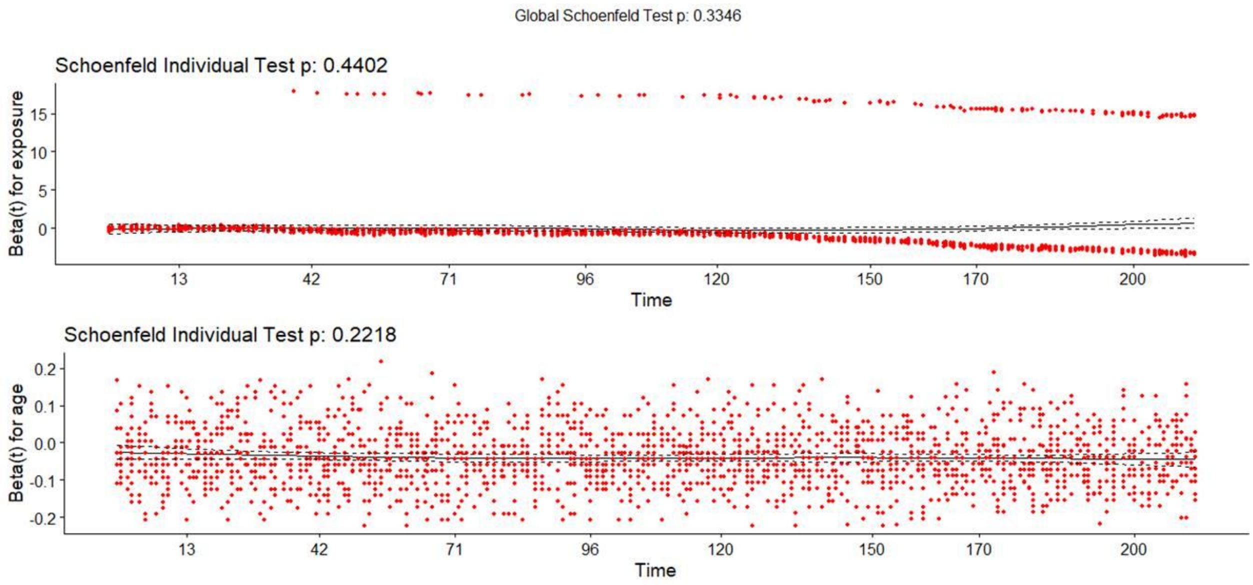

Period 2 Mass vaccination 2021-08-01 to 2021-12-31:

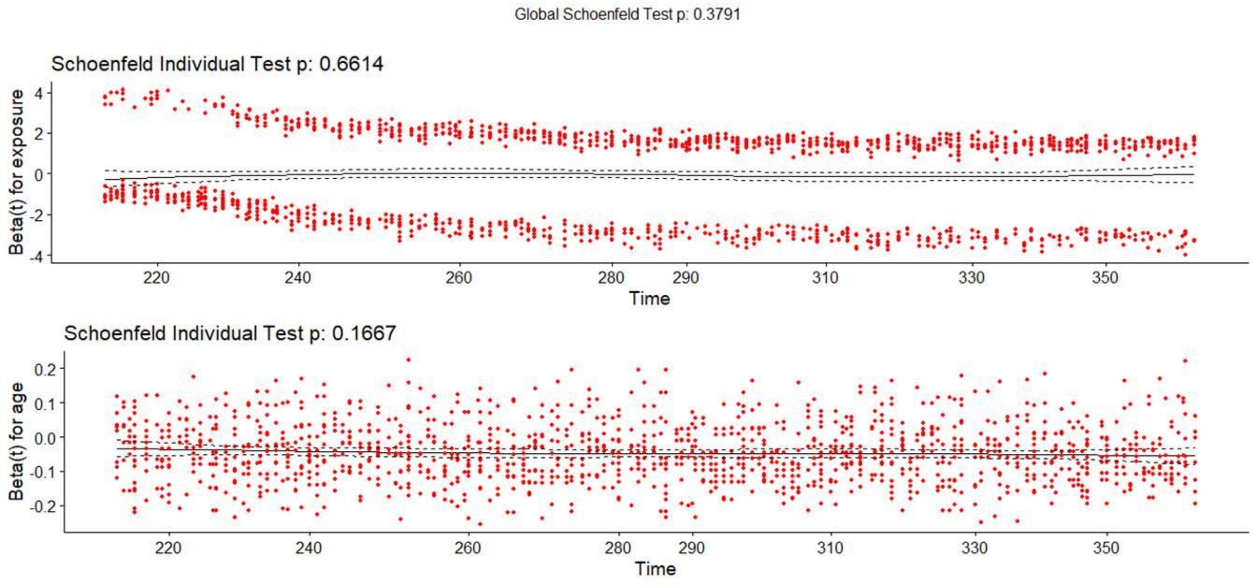

Period 3 Follow-up vaccination of unvaccinated individuals 2022-01-01 to 2023-09-30:

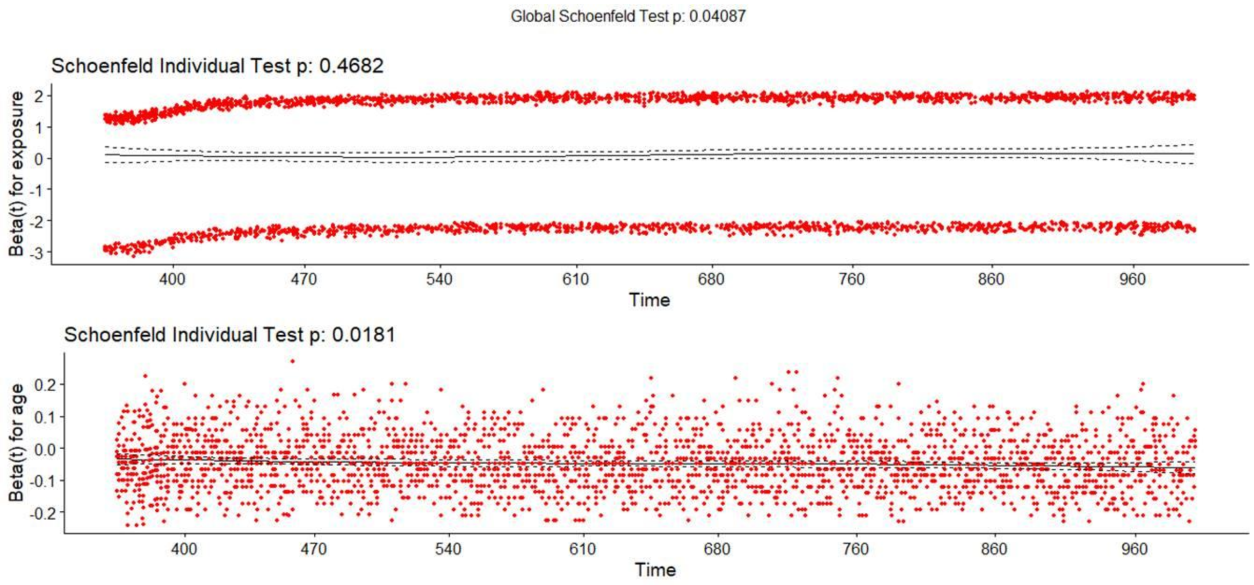

Figure S3.2 Schoenfeld tests of proportional hazards assumption for miscarriage. Residuals are scaled and plotted against time.

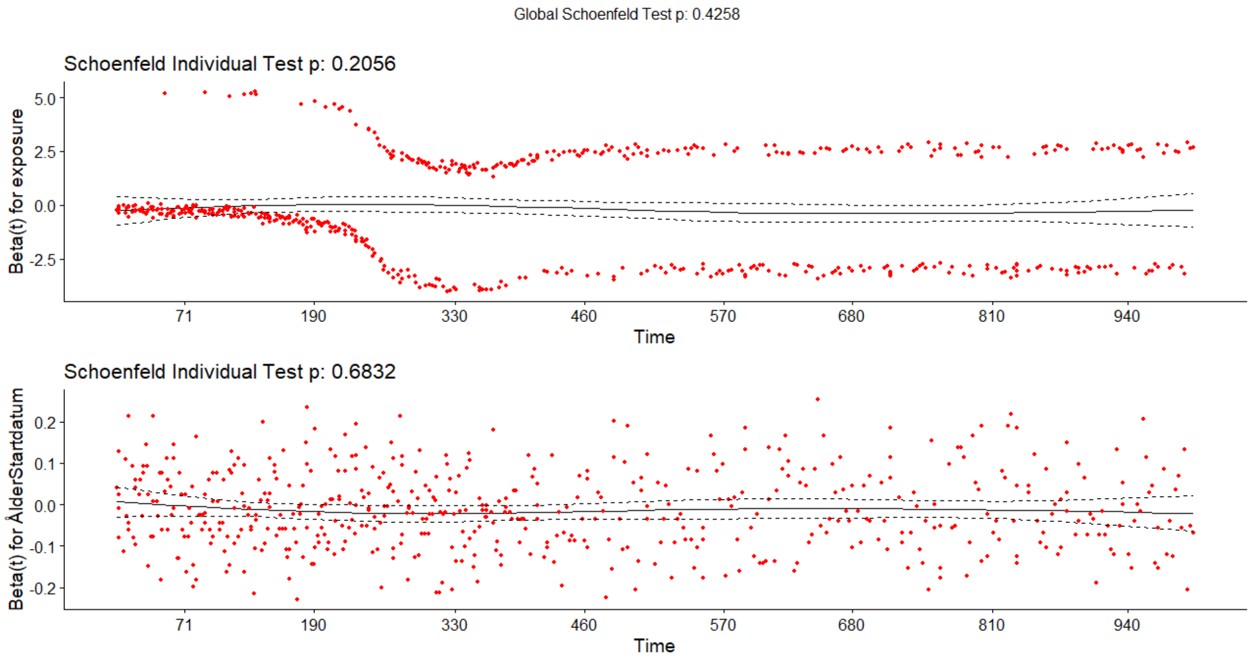

Figure S4 HR(t) for childbirth (280 days pregnancy) based on natural cubic splines.

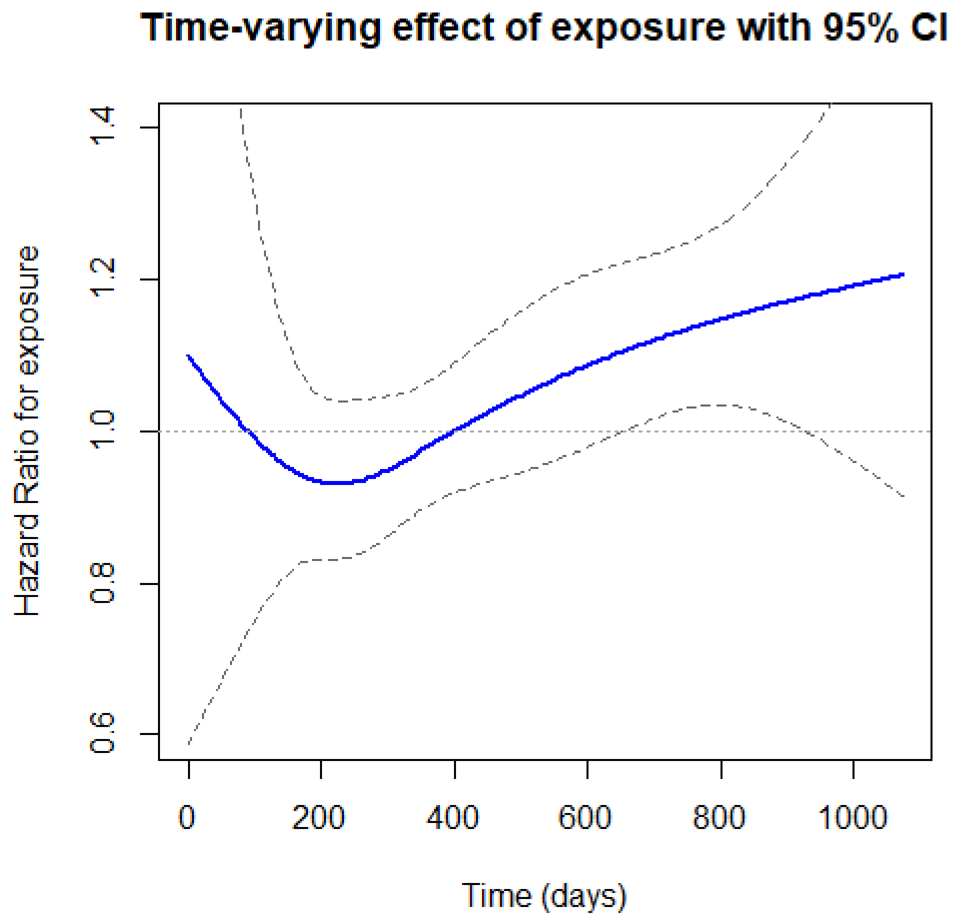

Table S5. Additional sensitivity analyses associated with vaccine dose 1.

The original algorithm (original result) adjusted
a) for exclusion of pre–dose 2 events in the time-split dataset (adjusted algorithm), and
b) including women with only one vaccine dose (adjusted algorithm including dose 1).

**HR (95% CI)**	**Childbirth (280 days pregnancy)**	**Childbirth (266 days pregnancy)**	**Miscarriage**
**Original result**	1.03 (0.97–1.09)	1.04 (0.98-1.11)	0.86 (0.70-1.05)
**Adjusted algorithm**	1.00 (0.94-1.06)	1.01 (0.95-1.07)	0.80 (0.66-0.97)
**Adjusted algorithm including dose 1**	1.02 (0.96-1.09)	1.02 (0.96-1.08)	0.90 (0.74-1.09)
Not censoring for dose 3			
Adjusted algorithm	*1.02 (0.97-1.08)*	*1.03 (0.97-1.09)*	*0.73 (0.61-0.87)*
Adjusted algorithm including dose 1	*1.05 (0.99-1.10)*	*1.04 (0.99-1.10)*	*0.82 (0.68-0.98)*
