# Supplementary material for: COVID-19 vaccination carries no association with childbirth rates in Sweden
Source: Commun Med (Lond). 2026 Jan 21;6:360. doi: 10.1038/s43856-026-01396-x (PMC13310814; doi:10.1038/s43856-026-01396-x)
Supplement: Supplementary file 3 — Description of Additional Supplementary files [file 43856_2026_1396_MOESM3_ESM.pdf]

Description of Additional Supplementary Files

File name: Supplementary Data

Description: Source Data for Figure 2
