# Supplementary material for: COVID-19 vaccination carries no association with childbirth rates in Sweden
Source: Commun Med (Lond). 2026 Jan 21;6:360. doi: 10.1038/s43856-026-01396-x (PMC13310814; doi:10.1038/s43856-026-01396-x)
Supplement: Supplementary file 1 — Transparent Peer Review file [file 43856_2026_1396_MOESM1_ESM.pdf]

nature portfolio

Peer Review File

COVID-19 vaccination carries no association with childbirth rates in Sweden

Corresponding Author: Professor Toomas Timpka

This file contains all reviewer reports in order by version, followed by all author rebuttals in order by version.

Version 0:

Reviewer comments:

Reviewer #1

(Remarks to the Author)
This study investigates whether COVID-19 vaccination was associated with a decrease in childbirth rates during the pandemic in Sweden, using health registry data and causal modeling to correct for bias. Among 59,773 women aged 18–45 in Jönköping County, Sweden, the authors find no significant association between vaccination and recorded childbirth, or miscarriage.

If I have understood correctly, the way in which the analysis has been done here considers individuals who are a) pregnant and b) not pregnant at time of vaccination as a single group, but the two groups in fact address different questions. Group a) is relevant to the safety of vaccination during pregnancy, whereas Group b) is relevant to the effect of vaccination on conception (and subsequently the effect of having been vaccinated on any subsequent pregnancy). Since these are two quite different biological phenomena, they would ideally be analysed separately. If this is not possible, the authors should at least make it clear in the text that the two are being analysed together. The abstract and introduction focus on fertility, so I initially assumed that the analysis was only of individuals who were not pregnant at time of vaccination – a better balance between discussion of fertility and pregnancy is needed.

“Data from women recorded as ≥20 weeks pregnant on 1 January 2021 were removed from this analysis.” This approach will retain some individuals who cannot have a miscarriage after vaccination. For example, someone who is 18 weeks pregnant on 1 January 2021, and is subsequently vaccinated on 1 April 2021 (at ~30 weeks) cannot have a miscarriage. Data from women vaccinated at ≥20 weeks pregnant should be removed from the dataset.

Supplementary Figure 2. I find this the most useful of the Figures supplied, as it illustrates the key point that there is no difference in the rate at which vaccinated versus unvaccinated women give birth. The authors should consider moving this into the body of the text.

Minor comments
Title. This title could be interpreted as implying an association between COVID-19 vaccination and childbirth decrease, although that is not what the authors report. Consider changing it.

“Epidemiological studies have not identified negative associations between fetal development or preterm birth and receipt of COVID-19 vaccination administered during any trimester (4,5).” Given the volume of research on the safety of COVID-19 vaccination in pregnancy, it would make sense to cite a meta-analysis, rather than two individual studies. I suggest Fernandez-Garcia, 2024, BMJ Global Health. Given that the study is also pertinent to effects of COVID-19 vaccination on fertility, it might also be worthwhile citing some of the research on this: to my knowledge there is one study of this in couples trying to conceive through intercourse: Wesselink, 2022, Am J Epidemiol. There are a number showing no effect in couples trying to conceive through assisted reproduction: again, I would cite a meta-analysis.

Figure 2. Trend lines are described in the figure legend, but do not appear in the Figure. A dashed black curve is described in the figure legend but not shown on the graph.

Reviewer #2

(Remarks to the Author)
The authors analyze covid vaccination among reproductive-aged women and childbirths that occur 266-280 days later. The hypothesis being tested is that if covid vaccination reduced fertility, births would fall approximately 9 months later.
Perhaps I'm misunderstanding the use of "knots" in the methods, which I associate with splines. Do the authors just mean the time frame of the study was limited to that period? Since individual-level data is available, how do the knots go beyond just timing of the vaccine for each person?
It seems like they never give a raw association. Age is a minimal adjustment, but it still makes me wonder. The authors mention using DAGs to identify confounders but only age was adjusted for?
The authors say there are no registries for induced abortion in the conceptual model, but also that induced abortions were excluded in the methods.
What is the gestational age of the included miscarriages?
What about later fertility? The assumption seems to be that the vaccine would just have an effect during pregnancy, but could it not theoretically cause a longer-term effect on fertility, if it has one on immunity?

Version 1:

Reviewer comments:

Reviewer #1

(Remarks to the Author)
My comments have been addressed to my satisfaction, given the allowed number of display elements in the main paper. I am happy to recommend the paper for publication.

Reviewer #2

(Remarks to the Author)
The authors have acceptably responded to the reviewers' comments.

Open Access This Peer Review File is licensed under a Creative Commons Attribution 4.0 International License, which permits use, sharing, adaptation, distribution and reproduction in any medium or format, as long as you give appropriate credit to the original author(s) and the source, provide a link to the Creative Commons license, and indicate if changes were made.
In cases where reviewers are anonymous, credit should be given to 'Anonymous Referee' and the source.
The images or other third party material in this Peer Review File are included in the article's Creative Commons license, unless indicated otherwise in a credit line to the material. If material is not included in the article's Creative Commons license and your intended use is not permitted by statutory regulation or exceeds the permitted use, you will need to obtain permission directly from the copyright holder.
To view a copy of this license, visit https://creativecommons.org/licenses/by/4.0/

Reviewers' comments:

Reviewer #1 (Remarks to the Author):

#1.1 This study investigates whether COVID-19 vaccination was associated with a decrease in childbirth rates during the pandemic in Sweden, using health registry data and causal modeling to correct for bias. Among 59,773 women aged 18–45 in Jönköping County, Sweden, the authors find no significant association between vaccination and recorded childbirth, or miscarriage.

If I have understood correctly, the way in which the analysis has been done here considers individuals who are a) pregnant and b) not pregnant at time of vaccination as a single group, but the two groups in fact address different questions. Group a) is relevant to the safety of vaccination during pregnancy, whereas Group b) is relevant to the effect of vaccination on conception (and subsequently the effect of having been vaccinated on any subsequent pregnancy). Since these are two quite different biological phenomena, they would ideally be analysed separately. If this is not possible, the authors should at least make it clear in the text that the two are being analysed together. The abstract and introduction focus on fertility, so I initially assumed that the analysis was only of individuals who were not pregnant at time of vaccination – a better balance between discussion of fertility and pregnancy is needed.
Authors' response: Thank you for the comment. Our aim was to study the total impact of COVID-19 vaccination on childbirth rates (Figure 1). The women included were not pregnant at the start of the observation period, i.e. pregnant and not pregnant women at the time of vaccination were therefore analyzed as a single group. This means that all causal effects of vaccination on childbirth were considered, including immune responses generated before and after conception.

We agree (and acknowledge in the paper) that the biological effects of vaccination on conception and development of the fetus are most likely to differ. However, our data do not permit to with certainty identify women pregnant and not pregnant, respectively, at the time of vaccination. This makes separating the effects challenging

The separate vaccine effects on conception and pregnancy, respectively, have still been considered and addressed in the study. Our analyses cover effects on conceptions (your research question B), although we lack data on failed conceptions and early spontaneous abortions. We clarify this fact in the text. Regarding vaccine effect on pregnant women (research question A), we addressed the risk for late spontaneous abortions in a separate analysis. This analysis did not show any significant effect of vaccination. We thus found no total effect of vaccination but acknowledge that a protective effect on one of the aspects (say, fertility) may hide a negative effect on the other aspect. Nonetheless, on request, we are willing to attempt to perform and report separate analyses.

#1.2 “Data from women recorded as ≥20 weeks pregnant on 1 January 2021 were removed from this analysis.” This approach will retain some individuals who cannot have a miscarriage after vaccination. For example, someone who is 18 weeks pregnant on 1 January 2021, and is subsequently vaccinated on 1 April 2021 (at ~30 weeks) cannot have a miscarriage. Data from women vaccinated at ≥20 weeks pregnant should be removed from the dataset.
Authors’ response: Miscarriages could be recorded in the health information system until week 22+0, thereafter lost pregnancies were recorded as still births. We have clarified that all women recorded as ≥22+0 weeks pregnant 1 January 2021 were removed from the analyses because they could not have a miscarriage recorded.

#1.3 Supplementary Figure 2. I find this the most useful of the Figures supplied, as it illustrates the key point that there is no difference in the rate at which vaccinated versus unvaccinated women give birth. The authors should consider moving this into the body of the text.
Authors’ response: We have discussed exchanging one of Figures 1-2 with Figure S2. However, we found that the two existing figures are needed for readers to rapidly understand the study. We have revised Figure S2 but prefer to keep it in the supplements due to that only two illustrations are allowed. If three illustrations would be allowed, we would of course add the figure to the main manuscript.

#1.4 Minor comments
Title. This title could be interpreted as implying an association between COVID-19 vaccination and childbirth decrease, although that is not what the authors report. Consider changing it.
Authors’ response: This is good point. The title has been revised.

“Epidemiological studies have not identified negative associations between fetal development or preterm birth and receipt of COVID-19 vaccination administered during any trimester (4,5).” Given the volume of research on the safety of COVID-19 vaccination in pregnancy, it would make sense to cite a meta-analysis, rather than two individual studies. I suggest Fernandez-Garcia, 2024, BMJ Global Health. Given that the study is also pertinent to effects of COVID-19 vaccination on fertility, it might also be worthwhile citing some of the research on this: to my knowledge there is one study of this in couples trying to conceive through intercourse: Wesselink, 2022, Am J Epidemiol. There are a number showing no effect in couples trying to conceive through assisted reproduction: again, I would cite a meta-analysis.
Authors’ response: We have added the reference and revised the language in the introduction.

Figure 2. Trend lines are described in the figure legend, but do not appear in the Figure. A

dashed black curve is described in the figure legend but not shown on the graph.
Authors' response: The figure legend has been revised according to the comments.

Reviewer #2 (Remarks to the Author):

#2.1 The authors analyze covid vaccination among reproductive-aged women and childbirths that occur 266-280 days later. The hypothesis being tested is that if covid vaccination reduced fertility, births would fall approximately 9 months later.
Perhaps I'm misunderstanding the use of "knots" in the methods, which I associate with splines. Do the authors just mean the time frame of the study was limited to that period? Since individual-level data is available, how do the knots go beyond just timing of the vaccine for each person?
Authors' response: To increase the transparency and reproducibility of the study, we have reanalyzed the data without using knots. Although theoretically justified, their use did not impact the results in a notable way. The manuscript has been updated to present the revised analyses.

#2.2 It seems like they never give a raw association. Age is a minimal adjustment, but it still makes me wonder. The authors mention using DAGs to identify confounders but only age was adjusted for?
Authors' response: Unadjusted associations have been added to the manuscript. Directed acyclic graphs were used as the basis for drawing a conceptual map of the assumptions about causal associations and confounding bias relating the exposure COVID-19 vaccination to the outcome childbirth (see revised Figure 1). Age and comorbidity were from this mapping identified as meaningful confounders. Ongoing comorbidity was assumed to 1) be associated with higher propensity to accept vaccines and 2) voluntarily abstain from pregnancy. As correction for comorbidity thus was presumed to diminish the possibility to observe a negative association between vaccination and childbirth, the analyses were corrected for age only. This is now explained in the manuscript.

#2.3 The authors say there are no registries for induced abortion in the conceptual model, but also that induced abortions were excluded in the methods.
Authors' response: Yes, this point was unclear in the submitted manuscript. Induced abortions are a special case. We have no access to these data for ethical reasons even though they are recorded (and therefore were included in the conceptual map). This is explained in the revised manuscript.

#2.4 What is the gestational age of the included miscarriages?
Authors' response: The gestational age of miscarriages included in the study ranged from the week the pregnancy was documented in the health information system (about 7 weeks) to 22+0 weeks. This is highlighted in the revised manuscript.

#2.5 What about later fertility? The assumption seems to be that the vaccine would just have an effect during pregnancy, but could it not theoretically cause a longer-term effect on fertility, if it has one on immunity?
Authors' response: We appreciate the comment. We thus aimed to study vaccine effects on conceptions and established pregnancies in the short-term. Both mrna vaccines and SARS-CoV-2 may theoretically induce autoimmune reactions with long-term impact on fertility. However, we find that the study aims, design, and data do not allow analysis and drawing meaningful conclusions about these long-term vaccine effects.
